# PhaeoNet: A Holistic RNAseq-Based Portrait of Transcriptional Coordination in the Model Diatom *Phaeodactylum tricornutum*

**DOI:** 10.3389/fpls.2020.590949

**Published:** 2020-10-16

**Authors:** Ouardia Ait-Mohamed, Anna M. G. Novák Vanclová, Nathalie Joli, Yue Liang, Xue Zhao, Auguste Genovesio, Leila Tirichine, Chris Bowler, Richard G. Dorrell

**Affiliations:** ^1^Institut de Biologie de l’Ecole Normale Supérieure (IBENS), Ecole Normale Supérieure, CNRS, INSERM, Université PSL, Paris, France; ^2^Department of Oceanography, Dalhousie University, Halifax, NS, Canada; ^3^Université de Nantes, CNRS, UFIP, UMR 6286, Nantes, France

**Keywords:** stramenopile, transcriptomics, sigma factors, aureochromes, epigenetics, chloroplast-mitochondria

## Abstract

Transcriptional coordination is a fundamental component of prokaryotic and eukaryotic cell biology, underpinning the cell cycle, physiological transitions, and facilitating holistic responses to environmental stress, but its overall dynamics in eukaryotic algae remain poorly understood. Better understanding of transcriptional partitioning may provide key insights into the primary metabolism pathways of eukaryotic algae, which frequently depend on intricate metabolic associations between the chloroplasts and mitochondria that are not found in plants. Here, we exploit 187 publically available RNAseq datasets generated under varying nitrogen, iron and phosphate growth conditions to understand the co-regulatory principles underpinning transcription in the model diatom *Phaeodactylum tricornutum*. Using WGCNA (Weighted Gene Correlation Network Analysis), we identify 28 merged modules of co-expressed genes in the *P. tricornutum* genome, which show high connectivity and correlate well with previous microarray-based surveys of gene co-regulation in this species. We use combined functional, subcellular localization and evolutionary annotations to reveal the fundamental principles underpinning the transcriptional co-regulation of genes implicated in *P. tricornutum* chloroplast and mitochondrial metabolism, as well as the functions of diverse transcription factors underpinning this co-regulation. The resource is publically available as PhaeoNet, an advanced tool to understand diatom gene co-regulation.

## Introduction

The biology of prokaryotic and eukaryotic cells is dependent on elaborate metabolic, regulatory and gene expression pathways, consisting of multiple interacting components. The successful operation of these pathways depend on the coordinated expression of genes that underpin them, which allow the stoichiometric assembly of their constituent components and enable discrete and appropriate regulatory responses to changes in physiological conditions (Gasch and Eisen, 2002; Teichmann and Babu, 2002). In prokaryotes and bacteria-derived genomes (e.g., “plastids” including chloroplasts and mitochondria) gene co-regulation is possible via the co-transcription of linked genes as part of the same transcriptional operon (Teichmann and Babu, 2002). In contrast, gene order plays a limited role in eukaryotic nuclear gene co-expression (Michalak, 2008), which depends instead on the simultaneous transcription, or transcriptional stabilization, of multiple discrete genomic loci. This may occur through common transcription factors (Teichmann and Babu, 2002; Reja et al., 2015); epigenetic modifications based around characteristic histone and DNA marks (Bird, 2002; Bártová et al., 2008); co-ordinated transcript processing events (Norbury, 2010); and the activity of small and long regulatory non-coding RNAs (Tsai et al., 2010; Kim and Sung, 2012).

The degree to which gene co-regulation is shared between different species is debated, with different studies identifying shared co-regulatory trends in between 8% (Teichmann and Babu, 2002) and 70% (Snel et al., 2004) of orthologous gene pairs between *Saccharomyces cerevisiae* (yeast) and *Caenorhabditis elegans* (nematode) genomes. Nonetheless, there is substantial merit to understanding gene co-regulation patterns in novel species. Since their origins over two billion years ago, the eukaryotes have radiated into a diverse range of different lineages, many of which are unicellular; and distantly related to model organisms in the animals, fungi and plants, with different underlying cellular biology and transcriptional dynamics (Walker et al., 2011). Identifying what gene co-regulation processes occur in microbial eukaryotes may allow us to better understand the biology underpinning the base of planetary food webs; and to better model the robustness of eukaryotic communities to environmental change.

The diatoms are a major group of predominantly marine algae, believed to be responsible for nearly one-fifth of total planetary photosynthesis (Field et al., 1998). Diatoms sit within the stramenopile supergroup, and are distantly related to animals, fungi and plants. Photosynthetic members of the stramenopiles, including diatoms, possess a chloroplast acquired through secondary endosymbiosis, unlike the primary plant chloroplast, which is of primary endosymbiotic origin (Walker et al., 2011). Previous genomic and functional investigations of model diatoms, for example *Phaeodactylum tricornutum*, have identified divergent features in their cellular biology, compared to more well-understood eukaryotic groups (Bowler et al., 2010). These include intricate metabolic connections between the diatom chloroplast, mitochondria and cytoplasm (Prihoda et al., 2012; Bailleul et al., 2015); and a wide range of different histone structural modifications (Veluchamy et al., 2013, 2015), many of which have not yet been detected in more established eukaryotic models.

Previously, microarray data from over 100 different conditions, including illumination regimes and pollutant stress (Osborn and Hook, 2013; Valle et al., 2014), have been generated from *P. tricornutum*; which have been assembled into a searchable interface, DiatomPortal that divides the *P. tricornutum* genome into 500 co-regulated gene clusters (Ashworth et al., 2016). Alongside this, a suite of RNA sequencing libraries exploring cellular responses to phosphorus, iron and nitrogen limitation have now been generated (Maheswari et al., 2010; Cruz de Carvalho et al., 2016; Smith et al., 2016; McCarthy et al., 2017) and inspecting these data may allow more precise integration of quantitative differences in transcript abundance than would be possible through microarray analyses. Furthermore, co-expression networks are a powerful tool for functional prediction and annotation of unknown genes in the absence of prior knowledge, which is the case for a significant number of genes in *P. tricornutum* (Rastogi et al., 2018). Co-expression networks can furthermore enrich our understanding of the more sparse co-expression networks generated for other marine algal species with secondary chloroplasts (principally, the distantly related diatom *Thalassiosira pseudonana*, the distantly related stramenopile *Nannochloropsis oceanica* and the haptophyte *Emiliania huxleyi*; Ashworth et al., 2016; Ashworth and Ralph, 2018).

Here, we use a tool of gene co-expression network analysis, WGCNA (Weighted Gene Correlation Network Analysis (Langfelder and Horvath, 2008; Guidi et al., 2016), to build PhaeoNet, an advanced tool for transcriptional understanding of the *P. tricornutum* genome. PhaeoNet is composed of 28 co-regulated gene modules, each with different expression dynamics. Considering the repartition of genes within these modules; functional, epigenetic and localization information from the third version annotation of the *P. tricornutum* genome (Phatr3; Rastogi et al., 2018); and annotated lists of diatom transcription factors (Rayko et al., 2010), we identify core features underpinning the transcriptional partitioning of diatom primary metabolism, including probable metabolic links between the diatom mitochondria and chloroplast; and dissect the diverse ranges of different transcriptional drivers of this co-regulation, notably in the case of chloroplast-targeted sigma factors. The raw data underpinning PhaeoNet have been made publically accessible via https://osf.io/42xmp.

## Materials and Methods

### Dataset Curation and Abundance Calculations

A total of 187 publically available RNA-seq datasets from *P. tricornutum*, generated from three studies exploring, respectively, phosphorus (Cruz de Carvalho et al., 2016), iron (Smith et al., 2016) and nitrogen (McCarthy et al., 2017) stress transcriptional responses, were collected from the sequence read archive (SRA)^1^ (Wheeler et al., 2006). The 182 libraries that passed through quality control steps, were included in the final version of the WGCNA performed, are named per their names respective studies in Supplementary Table 1, sheet 1. Data provided in the phosphate and nitrogen conditions were obtained using an Illumina Genome Analyzer (Bentley et al., 2008), while the iron study used SOLiD technology sequencing (Morey et al., 2013). *P. tricornutum* transcript IDs from each study were mapped to gene models based on the Phatr3 annotation of the genome (Supplementary Table 1, Sheet 2; Rastogi et al., 2018).

Raw data were reprocessed using FastQC version v0.11.5^2^. Low quality reads (Phred quality score below 20) were filtered-out using trim-galore version 0.5.0^3^. The remaining sequences were aligned to the reference genome with the software package STAR version 2.5.3a (Dobin et al., 2013) (STAR –outFilterMismatchNmax 2 –outFilterMultimapNmax 1000 –alignIntronMin 20 –alignIntronMax 2000). The iron data derived from the SOLiD technology were first mapped using the Life Technologies LifeScope software suitable for data from such technology. For homogeneity purposes, the reads were remapped using the pre-cited version of STAR.

Expression levels of individual genes were obtained using featureCounts version 1.6.1 (Liao et al., 2014). Quality checks of datasets were performed using methods provided in DESeq2 version 1.19.37 (Love et al., 2014), with a PCA projection and a hierarchical dendrogram using Spearman correlation between library-normalized gene counts (Glasser and Winter, 1961). These subsequent analyses and results visualizations were performed using R package version 3.4.4.

### Weighted Gene Correlation Network Analysis (WGCNA) and Network Visualization

The WGCNA R package (Langfelder and Horvath, 2008) was used to identify network modules from library-normalized gene expression values. First, a signed adjacency matrix (accepting oppositely correlated gene expression values to be clustered in the same modules) was obtained by calculating the pairwise Bi-weight mid-correlation coefficient from rij (Langfelder and Horvath, 2008), that represent expression values of genes i and j. A connectivity measure (k) per gene set was calculated by summing the connection strengths with other gene sets. Subsequently, the weighted adjacency matrix was obtained by raising the absolute value of the pairwise gene expression correlations to the soft-thresholding parameter β (Zhao et al., 2010). This achieved the scale-free topology criterion for WGCNA and typical for biological networks, emphasizing high correlations and minoring low ones, in which most nodes are not connected and only a few nodes are highly connected (Barabási, 2009).

The scale-free topology of PhaeoNet was evaluated by the Scale-Free Topology Fitting Index (*R*^2^), which was the square of the correlation between log[p(k)] and log(k). A β coefficient of 12 with *R*^2^ of 0.9 was used during the network building from the signed weighted adjacency matrix. The weighted adjacency matrix was finally used to calculate the Topological Overlap Matrix (TOM). Subsequently, modules were detected on the basis of the Topological Overlap measure using the following parameters: minModuleSize = 40 and mergeCutHeight = 0.25.

Graphical representations of the network were performed using Cytoscape (Shannon et al., 2003). All code used for the construction of PhaeoNet and interactive diagrams of each merged module are publically available through the following link: https://osf.io/42xmp.

### Biological Interpretation of Merged Modules

The distribution of *P. tricornutum* genes in each transcriptional module was compared to the distribution of orthologous gene models (Phatr2.0 genome annotation) in microarray-derived transcriptional clusters generated as part of the DiatomPortal project (Ashworth et al., 2016). Only gene models that showed a one-to-one gene mapping (i.e., gene models that were neither split or merged, but including gene models that were truncated or extended) between version 2 (Phatr2) and version 3 (Phatr3) annotations of the *P. tricornutum* genome (Bowler et al., 2008; Rastogi et al., 2018) were considered.

Biological functions within the merged modules were identified using gene functional annotations from the Phatr3 annotation of the *P. tricornutum* genome (Bowler et al., 2008; Rastogi et al., 2018). These included: GO terms, using the R package TopGO (Aibar et al., 2015); PFAM domains and biological processes (Rastogi et al., 2018); probable evolutionary affinities inferred by BLAST top hit analyses (Rastogi et al., 2018); histone and DNA modifications associated with cells grown in replete media (Veluchamy et al., 2013, 2015); Polycomb group protein marks (Zhao et al., 2020); and KEGG orthology predictions, obtained with BLASTkoala, Kofamkoala and GHOSTkoala servers (Moriya et al., 2007; Kanehisa, 2017; Aramaki et al., 2019; Kanehisa and Sato, 2020). *In silico* targeting predictions were performed for all N-complete protein sequences (i.e., protein sequences inferred to start in a methionine) within the dataset, using HECTAR (Gschloessl et al., 2008); ASAFind v2.0 (Gruber et al., 2015), in conjunction with SignalP v3.0 (Bendtsen et al., 2004); MitoFates, with a threshold detection value of 0.35 (Fukasawa et al., 2015; Dorrell et al., 2017); and WolfPSort, taking the consensus best-scoring prediction using animal, fungi and plant reference datasets (Horton et al., 2007). Enrichments in each category were analyzed both qualitatively/manually and by a simple pivot table and chi-squared test. Tabulated lists of all annotations are presented in Supplementary Table 2.

Core chloroplast and mitochondria-associated functions were assembled from a list of 524 KEGG ortholog numbers based on previously identified chloroplast and mitochondria functions in photosynthetic eukaryotes (Dorrell et al., 2017; Nonoyama et al., 2019; Novák Vanclová et al., 2020). Where multiple candidate proteins were detected, proteins were assigned to either the chloroplast, mitochondria, or dual chloroplast/mitochondria (Gile et al., 2015; Dorrell et al., 2017) based on *in silico* targeting predictions. Where no clear targeting predictions could be obtained, proteins were identified based on BLAST similarity to orthologous chloroplast- or mitochondria-targeted proteins from other algal and stramenopile species (Dorrell et al., 2017; Río Bártulos et al., 2018). Disregarding 135 query proteins coded by organellar genomes in diatoms (Yu et al., 2018) and 17 query proteins encoded by nuclear genes with no PhaeoNet module assigned, the final set comprised of 372 unique proteins targeted to the chloroplast and/or mitochondrion, encoded by nuclear genes that belong to one of the 28 merged modules. The main metabolic pathways and complexes and quantitative pathway associations, are presented in Supplementary Table 3.

A complete list of *P. tricornutum* transcription factors (TF) was assembled from a previous dataset (Rayko et al., 2010) and an updated list specifically of aureochromes (Banerjee et al., 2016), which were mapped to the version 3 genome annotation by BLASTp analysis. A total of 188 candidates, from 18 TF families (HSF, Myb, Zn_finger_C2H2, bZIP, Zn_finger_CCCH, bHLH, Sigma-70, Zn_finger_TAZ, CBF/NF, E2F-DP, CSF, Aureochrome, TRF, CCAAT-binding, AP2-EREBP, TAF9, CXC, Homeobox) corresponded to genes assigned to a PhaeoNet merged module (Figure 5 and Supplementary Table 4). Given that the regulation of gene expression by transcription factors play a key role in the growth and progression of the cell cycle, the distribution within merged modules genes implicated in the cell cycle (cyclins) and in light perception events (e.g., phytochrome, cryptochrome) were additionally investigated, as well as genes implied in transcription and histone-related processes (Figure 5 and Supplementary Table 4; Huysman et al., 2013; Annunziata et al., 2019).

### Phylogenetics

A tree of sigma factor proteins from *P. tricornutum* and orthologous diatom and non-diatom sequences was constructed using a pipeline adapted from previous studies (Dorrell et al., 2017; Rastogi et al., 2018). Briefly, the complete peptide sequences of each sigma factor protein (eight total) in the Phatr3 annotation of the *P. tricornutum* genome (Rastogi et al., 2018) were searched using BLASTp against a composite library consisting of 110 diatom genomes, MMETSP (Marine Microbial Eukaryote Transcriptome Sequencing Project, Keeling et al., 2014) and independent transcriptomes; and a reference set of 59 additional eukaryotic genomes, sampled from across the tree of life (Supplementary Table 5). Orthologs with an *e*-value of 10^–05^ or lower were extracted and searched against the complete protein sequences encoded within the Phatr3 annotation of the *P. tricornutum* genome via reciprocal BLASTp searches. Sequences which retrieved a single best hit against a *P. tricornutum* sigma factor protein were aligned using MAFFT v 8.0 (Katoh et al., 2002) under the –auto setting (BLOSUM62 matrix, gap open penalty 1.53, offset value 0) and the in-house alignment program in GeneIOUS v 10.0.9 (Kearse et al., 2012) using a more stringent set of conditions (65% similarity cost matrix, gap open penalty 12, gap extension penalty 3, two rounds of refinement). Poorly aligned or incomplete sequences were removed at each step. The 771 protein sequences retained were manually curated to retain a representative series of 86 diatom and non-diatom sequences related to each *P. tricornutum* sigma factor and trimmed using trimal with the –gt 0.5 setting (Capella-Gutiérrez et al., 2009) to yield a 453 aa alignment. The best-scoring tree topology was inferred from the alignment using RAxML v8.2, 100 bootstrap replicates and the PROTGAMMAJTT substitution model (Stamatakis, 2014); and MrBayes v3.2.7 over 600,000 generations, burnin fractions of 0.5 and the Jones amino acid substitution model (Huelsenbeck and Ronquist, 2001). Alignment and tree outputs are provided in Supplementary Table 5.

## Results and Discussion

### Construction of an Optimized WGCNA Gene Expression Dataset for *P. tricornutum*

**FIGURE 1:**
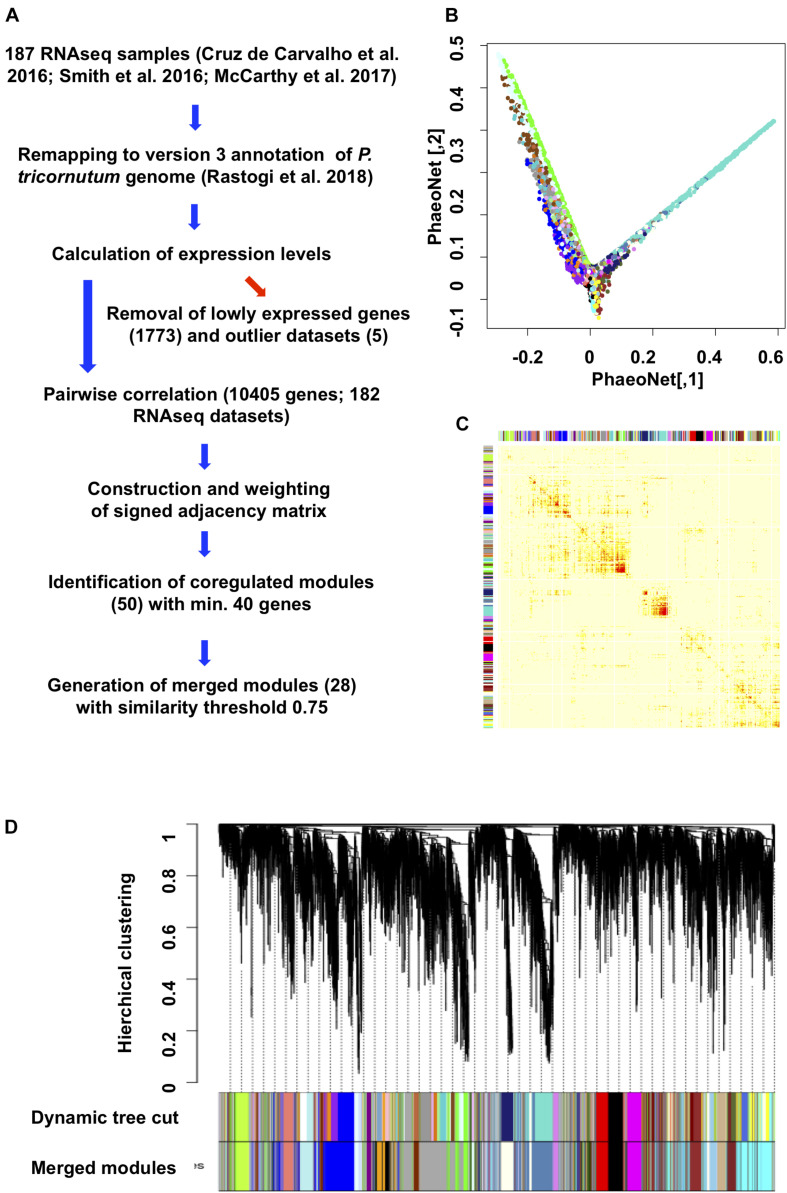
Construction and topology of PhaeoNet. **(A)** Workflow diagram of the steps performed to construct PhaeoNet. **(B)** A Multi-Dimensional Scaling (MDS) plot of PhaeoNet. The dots correspond to genes and the colors correspond to the WGCNA modules. The tips of the plot correspond to hub genes of PhaeoNet. **(C)** Heatmap plot showing the TOM supplemented by the WGCNA module colors prior to merging. **(D)** Gene dendrogram of all incorporated PhaeoNet genes, obtained by average linkage hierarchical clustering. The first color row underneath the dendrogram shows the WGCNA module assignment obtained by the Dynamic Tree Cut method. The bottom color row shows the merged modules based on a correlation threshold of 0.75.

We harnessed 187 publically available RNAseq datasets derived from diverse physiological conditions and genotypes (Cruz de Carvalho et al., 2016; Smith et al., 2016; McCarthy et al., 2017) to build an integrative model of gene co-regulation for the model diatom species *P. tricornutum* (Figure 1A and Supplementary Table 1, sheet 1). We chose to build a dataset focusing on one species only, as even closely related diatom species may contain very different protein orthogroups (Parks et al., 2018; Sato et al., 2020) and even orthologous proteins may perform different physiological functions between different diatom species, with presumably different co-regulatory dynamics (Lampe et al., 2018). *P. tricornutum* was selected as a model system for this study as vastly greater amounts of gene expression data have been generated for this species than any other marine alga (Ashworth and Ralph, 2018); and as its genome annotation (currently in third version form and verified by comparison to over forty RNAseq libraries generated under varied conditions, Rastogi et al., 2018) is arguably the most complete of any alga known, allowing unprecedented insight into protein diversity, including variant protein forms generated by alternative splicing, protein sub-cellular localization and epigenetic modifications. The use of RNAseq data for this analysis allows us to advance on previous (e.g., microarray-based, Ashworth et al., 2016) analyses by allowing us to consider absolute rather than relative changes in expression levels between different datasets, and therefore exclude distorting effects caused by low absolute levels of the expression of specific genes in the *P. tricornutum* genome.

We optimized our data through several key pre-processing steps, for example removing batch effects (Supplementary Figure 1A) and five samples showing strong outlier effects (exemplar shown in Supplementary Figure 1B) prior to network construction, retaining 182 datasets for the final network construction. We also excluded genes that were found to be lowly expressed (median expression < 10 reads) in all inspected conditions, retaining 10,650/12,177 genes in the Phatr3 annotation (Rastogi et al., 2018) of the *P. tricornutum* genome (Supplementary Table 1, sheet 2). All pairwise gene correlations were calculated and then converted into connectivity strengths by raising their values to the power β = 12 for PhaeoNet. This power makes it possible to work in a scale-free condition and to avoid weak correlations (Supplementary Figure 2).

By applying the dynamic tree cut function on the dendrogram obtained by a hierarchical clustering with the method average, we identified 50 WGCNA modules with similar connection force profiles (Figures 1B,C). This was reduced to a subset of 28 merged modules with internal correlations above 0.75 (Figure 1D, Supplementary Table 1, sheet 2; and Supplementary Figure 3), in accordance with other WGCNA studies (Langfelder and Horvath, 2008; Zhao et al., 2010) and following validation by cross-referencing to independently derived gene co-regulation datasets for *P. tricornutum* (described below). The final version of PhaeoNet showed good overall cohesion within the merged modules, as inferrable by multi-dimensional-scaling projection (Figure 1C) and correlation heatmaps of gene co-expression interconnectedness (Figure 1B).

**FIGURE 2:**
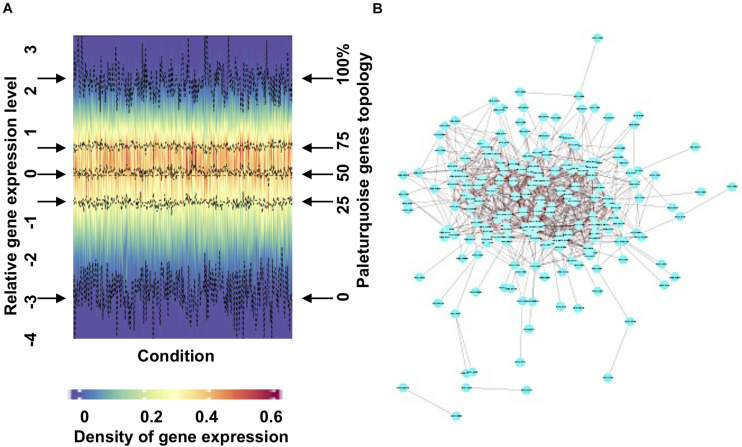
Visualization and analysis of an exemplar PhaeoNet merged module (paleturquoise). **(A)** Density heatmap of all genes assigned to the paleturquoise merged module. The y-coordinate positions in the graph relate to density distribution of gene expression in each sample (shown on the left *y*-axis); the four middle dashed lines (indicated by horizontal arrows on either side of the graph) correspond to the median, first and third quantiles (shown on the right *y*-axis). The majority of the genes in this specific module show limited variation in expression profiles over different conditions samples. **(B)** A topological representation of connectedness within the paleturquoise merged module, visualized with Cytoscape (version 3.6.1). Each node represents a gene. Edges represent pairwise correlations between genes. The network shows all the paleturquoise module genes with a correlation value over a threshold of 0.20.

We present an exemplar merged module output (paleturquoise) in Figure 2. A density heatmap, divided vertically by condition and horizontally by gene expression profile, shows a cohesive module as illustrated by stable values of first quantile, median, and third quantile values (Figure 2A) and is defined by high levels of expression across the majority of the conditions explored (Supplementary Figure 3B). Cytoscape (Shannon et al., 2003) visualization of the network with a correlation threshold of 0.2 (Figure 2B) demonstrates that the paleturquoise merged module is highly connected, showing a cluster of hub genes with high connectivity located in the central part of the network and only a small number of genes with limited connectivity. We provide detailed expression and Cytoscape data for each PhaeoNet merged module via https://osf.io/42xmp.

### PhaeoNet Merged Modules Show Concordance With Microarray Co-regulation Data

We tested the reproducibility of our assignations, which may be considered as an independent measure of their robustness, by comparing the repartition of all *P. tricornutum* genes assigned to a PhaeoNet merged module with their corresponding distributions in 500 co-regulated clusters previously assembled from microarray data within the DiatomPortal server (Ashworth et al., 2016; Supplementary Figure 4A and Supplementary Table 2). Across 7,751 assessable genes with both PhaeoNet and DiatomPortal assignations, we identified 4,127 (53%) that occurred in the same PhaeoNet merged module as another gene with the same DiatomPortal cluster assignation; and 2,751 genes (35%) that occurred within the single PhaeoNet merged module incorporating the greatest number of genes from the same DiatomPortal cluster. Both of these frequencies were judged to be significantly greater than expected through a random distribution (*P* = 0, one-tailed chi-squared test), suggesting strong concordance between both datasets.

From the 461 (83%) DiatomPortal clusters for which we could identify corresponding PhaeoNet merged modules, 369 (80%) were preferentially distributed in one PhaeoNet merged module only, with the greatest number of clusters associated with the darkgray merged module (79 clusters), blue (46 clusters) and cyan (44 clusters) merged modules, reflecting the greater size of each merged module (Supplementary Figure 4A and Supplementary Table 2). No DiatomPortal clusters were found to be incorporated preferentially into the bisque4, darkmagenta, greenyellow, gray, lightsteelblue1 and mediumpurple3 PhaeoNet merged modules. It is possible that these merged modules represent transcriptional networks not visualized within DiatomPortal due to the different source datasets, generated using different techniques (e.g., microarray versus RNAseq data, assembled with hierarchical clustering versus WGCNA; Ashworth et al., 2016), which may influence what genes are inferred to be coexpressed using each analysis.

We also verified the number of associations independently found between pairs of genes in DiatomPortal clusters and PhaeoNet modules generated with independent merging thresholds, as an independent test of the appropriateness of our selected 0.75 merging threshold (Supplementary Figure 4B). We found greater concordance between DiatomPortal and PhaeoNet modules generated with a 0.75 merging threshold, as in our methodology, than in unmerged WGCNA modules, or modules merged with higher (0.8) or lower (0.7) threshold values (Supplementary Figure 4B).

### Different PhaeoNet Merged Modules Perform Different Biological Activities in the *P. tricornutum* Cell

**FIGURE 3:**
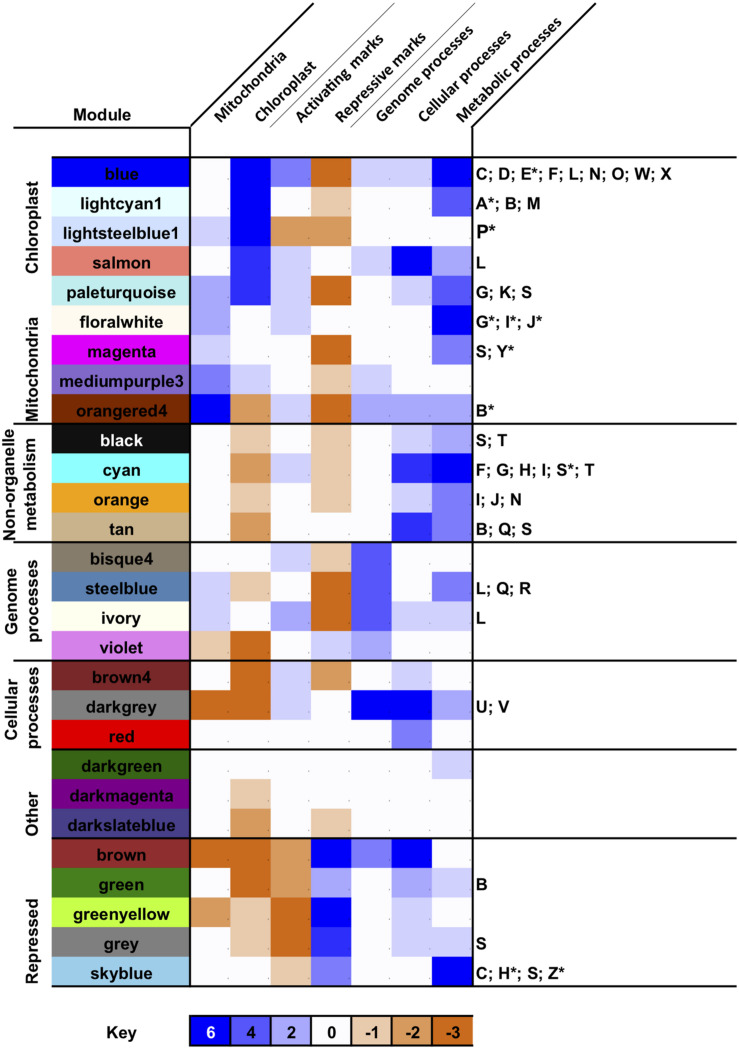
Biological properties associated with PhaeoNet merged modules. This Figure provides an overview of enrichments of different organelle targeting (Horton et al., 2007; Gschloessl et al., 2008; Fukasawa et al., 2015; Gruber et al., 2015), epigenetic (Veluchamy et al., 2013, 2015; Zhao et al., 2020), evolutionary (Rastogi et al., 2018) and KEGG pathway annotations (Kanehisa, 2017) enriched in merged modules. The first seven (shaded) columns provide a score for different conditions, aggregated from chi-squared P-values of multiple enrichment predictors (defined beneath): enrichments in each condition carry a score of +1 if significant to *P* < 0.05 and +2 if significant to *P* < 10^–05^; and depletions in each condition carry a score of –1 if significant to *P* < 0.05 and –2 if significant to *P* < 10^–05^, assessed by chi-squared test against a null hypothesis of a random distribution of these features across all genes assigned to a PhaeoNet merged module. The final column lists all metabolic pathways enriched to *P* < 0.05, or *P* < 10^–05^ (asterisked) for each merged module, assessed by chi-squared test as above. Verbose outputs for each set of conditions are provided in Supplementary Figure 5. Additional annotations, e.g., enrichments in inferred evolutionary origins of each merged module, are provided for user exploration in Supplementary Table S2.

Next, we profiled the predominant biological activities associated with each merged module by calculating enrichment scores for different functional, subcellular targeting and evolutionary annotations across the *P. tricornutum* genome (Rastogi et al., 2018; Figure 3 and Supplementary Figure 5). A full set of protein annotations *P. tricornutum*, including PhaeoNet module assignations, inferred functions, predicted localization and inferred evolutionary origin, is provided for user exploration in Supplementary Table 2.

We identified seven major subsets of merged modules with different biological properties. The first subset consists of merged modules (blue, lightcyan1, lightsteelblue1 and salmon) associated with the chloroplast [either genes encoding chloroplast-targeted proteins, inferred with ASAFind (Gruber et al., 2015) or HECTAR (Gschloessl et al., 2008), or of inferred red algal origin in a previous BLAST top hit analysis of the *P. tricornutum* genome (Rastogi et al., 2018)]. We included proteins of red algal origin as an independent estimator of chloroplastic origin, as the vast majority of red algal protein in *P. tricornutum* likely derive from the diatom chloroplast endosymbiont (Dorrell et al., 2017) and to allow us to detect chloroplast-associated proteins that elude *in silico* targeting prediction (Nonoyama et al., 2019; Schober et al., 2019). These merged modules were also enriched (as inferred with KEGG analysis (Kanehisa, 2017) in genes related to photosynthesis, carbon-fixation and core biosynthetic pathways (e.g., amino acid and pigment biosynthesis) associated with diatom chloroplasts (Figure 3 and Supplementary Figure 5; Nonoyama et al., 2019). Nearly all of the merged modules within this subset were enriched in activating histone marks (e.g., H3K9Ac and H3K14Ac) and depleted in repressive marks (e.g., H3K9me2 and H3K27me3) in cultures grown under replete media conditions (Figure 3 and Supplementary Figure 5; Veluchamy et al., 2015; Zhao et al., 2020), consistent with high levels of expression. Each of the chloroplast-enriched modules contained enrichments in different KEGG functions (discussed below), although only one of these modules (blue) was enriched in proteins containing at least one KEGG annotation (Supplementary Figure 5); and, in any case, all merged modules contain substantial numbers (between 18%, bisque4; and 54%, violet).

A second parallel set of merged modules (floralwhite, magenta, mediumpurple3, and orangered4), which was also found to be enriched in activating histone marks, was enriched in genes encoding mitochondria-targeted proteins (inferred with MitoFates, HECTAR and WolfPSort (Horton et al., 2007; Gschloessl et al., 2008; Fukasawa et al., 2015) and mitochondria-associated functions (e.g., oxidative phosphorylation and pyruvate metabolism; Figure 3 and Supplementary Figure 5). Of note, the paleturquoise merged module was uniquely enriched in genes encoding both chloroplast and mitochondria-targeted proteins, suggesting a probable hub between both organelle functions (Figure 3).

We identified three further subsets of merged modules that were enriched in cytoplasmic or nuclear processes involved in metabolism (black, cyan, orange, and tan); genome-associated processes including transcription, translation and genome repair (bisque4, steelblue, ivory and violet); or cellular processes including protein modification, protein trafficking and the cell cycle (brown4, darkgray, and red; Figure 3 and Supplementary Figure 5). Certain merged modules contained a mixture of genes encoding both metabolic and non-metabolic proteins: amongst other examples, the steelblue merged module was found to be enriched both in genes encoding proteins associated with ribosome and tRNA biogenesis and also in genes encoding enzymes involved in purine and pyrimidine metabolism, suggesting a probable transcriptional coordination of nucleotide biosynthesis to translational activity in *P. tricornutum* cells (Figure 3 and Supplementary Figure 5). A sixth subset of merged modules (darkgreen, darkmagenta and darkslateblue) showed no obvious enrichment in any KEGG function or organelle localization, except for a possible enrichment in peroxisomal functions in the darkgreen merged module (Davis et al., 2017).

The final merged module subset (brown, green, greenyellow, gray, and skyblue) was uniquely enriched in repressive histone marks and depleted in activating histone marks, in cultures grown on replete media (Figure 3; Veluchamy et al., 2015). These merged modules may either be constitutively repressed in *P. tricornutum* cells, or might lose their repressive histone marks and be expressed in alternative conditions to the replete culture conditions in which the epigenetic datasets were collected (Zhao et al., 2020). We noted that the greenyellow merged module, for example, was enriched in proteins with at least one KEGG annotation; and the skyblue merged module was found to be significantly enriched in genes encoding proteins involved in carbon fixation, the TCA cycle and propionate metabolism (Figure 3 and Supplementary Figure 5). Further studies of the epigenetic marks associated with these modules, including under physiological conditions in which they are most highly expressed (Supplementary Figure 3) will be necessary to determine under what circumstances the genes they contain make significant contributions to *P. tricornutum* biology.

### PhaeoNet Merged Modules Reveal Transcriptional Co-regulation in *P. tricornutum* Chloroplast and Mitochondrial Metabolism

**FIGURE 4:**
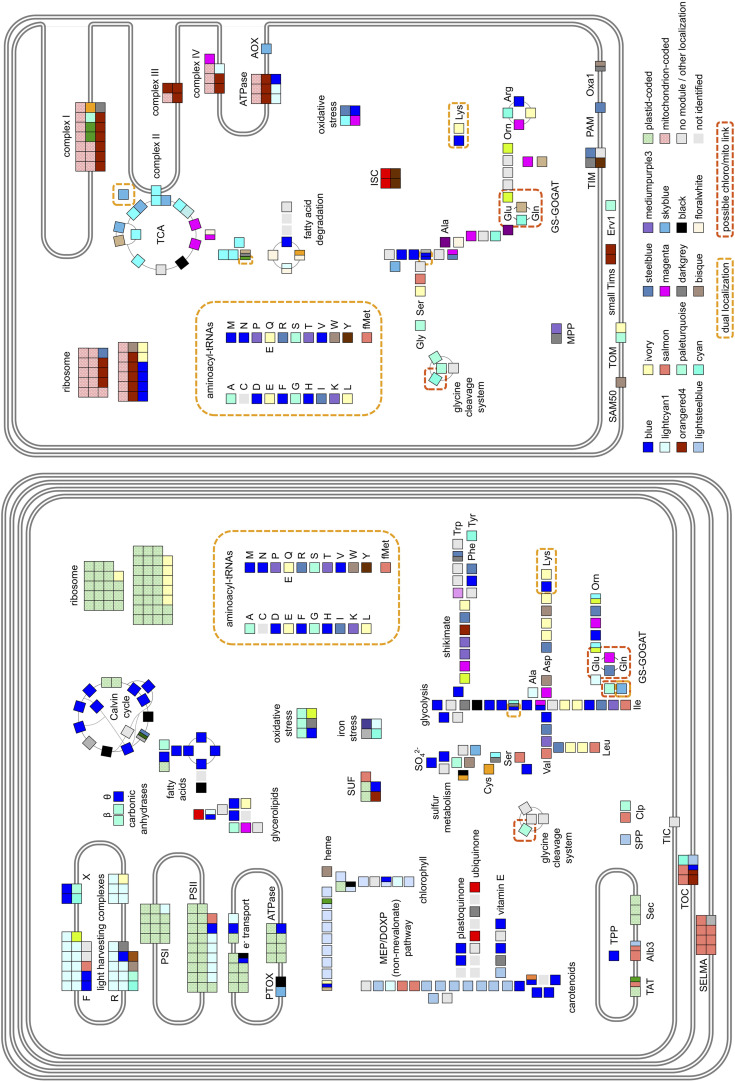
Main metabolic pathways and functional complexes of *P. tricornutum* plastid **(left)** and mitochondrion **(right)** and their composition in regard to PhaeoNet merged modules. Each square represents a gene encoding a protein identified either from N-terminal targeting predictions to function in the chloroplast or mitochondrion. Clusters of adjacent squares pertain to genes encoding different components of a specific multi-unit enzyme or complex; and split squares pertain to genes encoding functional homologs of one specific protein. The assigned merged modules are indicated as their respective colors, with the 16 most abundant merged modules shown in the legend. Additionally, proteins coded in organellar genomes (Oudot-Le Secq et al., 2007; Oudot-Le Secq and Green, 2011; Yu et al., 2018) are shown as dotted green or red; proteins for which chloroplast- or mitochondria-targeted isoforms or merged modules could not be assigned are shown as light gray; and enzymatic steps not identified in the genome are shown as light gray squares without borders. Dual-localized proteins (Gile et al., 2015; Dorrell et al., 2017) are marked by checkered yellow boxes; while orange boxes highlight potential connection points between the two organelles. Abbreviations are as follows: CAs, carbonic anhydrases; MEP/DOXP, mevalonate and non-mevalonate pathways for isoprenoid biosynthesis; SUF, iron-sulfur complex assembly; MPP,/TPP/SPP, mitochondrial, thylakoid and stromal processing peptidases; TAT, twin-arginine-dependent thylakoid protein import pathways; AOX/PTOX, mitochondrial and chloroplast alternative oxidases; TCA, Citric Acid cycle; Orn, ornithine; GCS, glycine shuttle; GS-GOGAT, glutamine synthetase/glutamate synthase shuttle. Detailed enzyme distributions for each pathway are shown in Supplementary Figure 6.

Having noticed specific biases in the distribution of mitochondria- and chloroplast-targeted proteins within our dataset and given the distinctive organelle metabolism noted in diatoms compared to plants (Kroth et al., 2008; Nonoyama et al., 2019; Smith et al., 2019), we wished to identify which key chloroplast and mitochondrial functions are revealed by PhaeoNet to be transcriptionally coordinated with one another. We searched the distribution of 372 manually curated nuclear-encoded proteins with known chloroplast- and mitochondria-associated functions and localizations (Figure 4, Supplementary Figure 6, and Supplementary Table 3). At least one gene encoding one such protein of each merged module was present in this set, however, only 12 merged modules contained more than 10 genes and amounted to 83% of the set.

The most abundantly represented merged module (blue, 69 chloroplast or mitochondrial occurrences) was clearly associated with genes encoding chloroplast anabolic reactions, containing enzymes associated with the Calvin–Benson–Bassham (CBB) cycle, chloroplast-targeted glycolysis/gluconeogenesis (Kroth et al., 2008) and fatty acid synthesis (Maréchal and Lupette, 2020), along with theta-class carbonic anhydrases that mediate biophysical carbon concentrating mechanisms in diatom chloroplasts (Figure 4 and Supplementary Figure 6; Kikutani et al., 2016; Nonoyama et al., 2019). The blue merged module additionally contained genes encoding chloroplast-targeted proteins implicated in photoprotection, including the diatom xanthophyll cycle (e.g., Phatr3_J51703 encoding violaxanthin de-epoxidase; Frommolt et al., 2008; Dautermann and Lohr, 2017), tocopherol synthesis (e.g., Phatr3_J20470, encoding tocopherol cyclase; Dłużewska et al., 2016; Nonoyama et al., 2019) and two genes (Phatr3_J27278 and Phatr3_J44733) encoding LhcX-class chlorophyll-binding proteins, associated with high- and low-light adaptation responses in diatoms (Supplementary Tables 2, 3; Taddei et al., 2016; Buck et al., 2019).

Genes encoding photosynthetic metabolism enzymes were concentrated in the lightcyan1 (41 occurrences) and lightsteelblue1 merged modules (19 occurrences). The lightcyan1 merged module included genes encoding LhcF-, LhcR-, and chlorophyll a/b-binding proteins, which are typically considered not to be involved in light stress responses (Gundermann et al., 2013; Büchel, 2015) and nucleus-encoded subunits of photosystems I, II and cytochrome c_6_ (Grouneva et al., 2011; Roncel et al., 2016); whereas the lightsteeblue1 merged module contained the majority of genes involved in diatom chlorophyll and isoprenoid synthesis (Bertrand, 2010; Cihlar et al., 2016). We noted the presence of two genes encoding enzymes involved in pigment biosynthesis, respectively carotenoids (Phatr3_J21829, encoding 2-C-methyl-D-erythritol 4-phosphate cytidylyltransferase) and chlorophyll (Phatr3_J30690, encoding 3,8-divinyl protochlorophyllide-a 8-vinyl reductase (Wang et al., 2010) in the lightcyan1 merged module (Supplementary Figure 6). We also noted the presence of the gene Phatr3_J47674 encoding the iron stress-induced protein ISIP3 within the lightcyan1 merged module, which may point to a functional role for this protein in chloroplast photosystem assembly (Supplementary Figure 6; Allen et al., 2008; Chappell et al., 2015).

Genes encoding mitochondrial respiratory chain proteins were concentrated toward the orangered4 merged module (27 occurrences), whereas, genes encoding TCA cycle enzymes were concentrated toward the cyan merged module (34 occurrences). The orangered4 merged module also contained large numbers of genes encoding mitochondrial ribosomal proteins, which may relate to redox-state dependent regulation of mitochondrial biogenesis pathways (Allen, 2003). In contrast, most genes encoding chloroplast biogenesis-related proteins were identified in separate PhaeoNet merged modules to genes encoding proteins of the photosystem core, with significant enrichments of genes encoding chloroplast ribosomal proteins in the ivory merged module (otherwise enriched in chloroplast branched-chain amino acid and lysine biosynthesis (Bromke, 2013). It remains to be determined to what extent the expression of the chloroplast- and mitochondrial-genomes of *P. tricornutum* are regulated in response to the redox state, versus metabolic fluxes experienced in both organelles.

Finally, we considered the repartition of functionally uncharacterized, but conserved domains across chloroplast-targeted proteins in our dataset, focusing on DUFs (Domains of Unknown Function). We found 30 chloroplast-targeted proteins containing at least one DUF and 8 DUFs assigned to at least two chloroplast-targeted proteins (Supplementary Table 3, Sheet 2). Amongst these recurrent chloroplast-associated DUFs were two examples (DUF1995 and DUF3493), which have previously been implicated to function in photosystem assembly within thylakoid membranes (Chi et al., 2012; Bohne et al., 2016; Li et al., 2019). Both of these DUFs were found amongst chloroplast-targeted proteins in the paleturquoise merged module (Phatr3_J38149, Phatr3_J40136, and Phatr3_J46926, containing DUF1995; and Phatr3_EG02444, containing DUF3493); in the blue module (Phatr3_J44212, containing DUF1995; and Phatr3_J45569, containing DUF3494); and DUF1995 furthermore occurred in chloroplast-targeted proteins in the lightcyan1 (Phatr3_J44529) and lightsteelblue (Phatr3_J40199) modules (Supplementary Table 3, Sheet 2). Each of these modules are enriched in different chloroplast-targeted metabolism pathways (Figures 3, 4), suggesting complex connections between the regulation of chloroplast anabolism and photosystem assembly.

Amongst the other DUFs associated with more than one chloroplast-targeted protein were DUF814, which is implicated in RNA quality control and amongst *P. tricornutum* chloroplast-targeted protein includes one (Phatr3_J45207, within the paleturquoise module) with some structural homology to a ferrous iron transporter (Maxwell Burroughs and Aravind, 2014); and DUF563, which contains a carbohydrate-active domain (Park et al., 2010) and includes at least one chloroplast-targeted protein (Phatr3_EG00581) within the blue module, otherwise implicated in chloroplast carbon metabolism (Figures 3, 4). It remains to be determined if either of these proteins has novel functions, e.g., respectively in iron status sensing or in the diversification of carbohydrate metabolism in the *P. tricornutum* chloroplast, via the generating and phenotyping of mutant lines.

### PhaeoNet Merged Modules Identify Complex Crosstalk between the Chloroplast and Mitochondrion in *P. tricornutum*

Previously, intricate metabolic connections have been observed between *P. tricornutum* chloroplasts and mitochondria, which are distinctive to those found in plants (Prihoda et al., 2012; Bailleul et al., 2015; Broddrick et al., 2019; Murik et al., 2019). We wished to determine which of these connections were visible within our data, noting multiple, transcriptionally independent connections between the predicted proteomes of chloroplasts and mitochondria in PhaeoNet data (highlighted in Supplementary Figure 6). These included the presence of genes encoding chloroplast-targeted protein import subunits (e.g., Phatr3_J32195 encoding Tic20, Phatr3_EG02421 encoding Tic21) within the otherwise predominantly mitochondrial orangered4 merged module and the presence of large numbers of amino-acyl tRNA synthetase genes (which are typically dual-targeted to the chloroplasts and mitochondria in diatoms (Gile et al., 2015; Dorrell et al., 2017, 2019) in the otherwise chloroplast-associated blue merged module.

We furthermore noted the presence of multiple chloroplast-targeted proteins associated with chloroplast division (e.g., Phatr3_J34093, Phatr3_J42361, and Phatr3_J14995, encoding FtsZ-type division proteins) in the blue module, potentially linking the synthesis of chloroplast and mitochondrial tRNAs to chloroplast replication. A further two proteins implicated in chloroplast replication (e.g., Phatr3_J21455, encoding a dynamin-related DRPB85-class protein and Phatr3_J14426, encoding a further FtsZ protein)—were found in the darkgray module, which was also populated by proteins involved in mitochondrial protein import (MPP, TIM, OXA1; Supplementary Table 3), suggesting probable links between chloroplast and mitochondrial biogenesis. Of note, at least two of the FtsZ proteins (Phatr3_J34093, within the blue module and Phatr3_J14426, within the darkgray module) were inferred to possess both chloroplast and mitochondrial-targeting sequences, underpinning the likely coordination of biogenesis of both organelles (Supplementary Table 3). This coordination may underpin the close topological associations and synchronized division cycles observed between the *P. tricornutum* mitochondrion and chloroplast observed *in vivo* (Tanaka et al., 2015; Dorrell and Bowler, 2017).

Alongside these more general links, we identified specific points of co-regulation between each organelle. The paleturquoise merged module, as the only merged module found to be enriched in both chloroplast and mitochondria functions (Figure 3) was of particular interest and contained genes encoding enzymes participating in several different chloroplast and mitochondria metabolic pathways. These included genes for mitochondria-targeted glycine dehydrogenase (Phatr3_J22187) and serine hydroxymethyltransferase (Phatr3_J32847) and a gene for a chloroplast-targeted dihydrolipoamide dehydrogenase (Phatr3_J30113), which participate (as part of the glycine shuttle) in metabolic recycling of 2-P-glycolate produced through photosynthesis (Supplementary Figure 6; Zheng et al., 2013; Davis et al., 2017). The paleturquoise merged module additionally contains a gene encoding mitochondria-targeted malate dehydrogenase (Phatr3_J54082), which may additionally participate in the photorespiratory metabolism of glycolate by allowing the recycling of mitochondrial serine (via pyruvate) in the TCA cycle (Davis et al., 2017; Broddrick et al., 2019). Genes encoding at least three further plastidial oxidative stress-related proteins (Phatr3_J12583, encoding Fe-Mn family superoxide dismutase; Phatr3_J45252, encoding a plastidial thioredoxin; and Phatr3_J31436, encoding a plastidial ortholog of peroxisomal membrane protein 2, Davis et al., 2017; Dorrell et al., 2017) belong to the paleturquoise merged module, underlining its importance in oxidative stress responses.

Genes encoding both glutamine synthase (GS) and glutamate synthase/glutamine oxoglutarate aminotransferase (GOGAT), which have distinct plastidial and mitochondrial homologs in *P. tricornutum* (Broddrick et al., 2019; Smith et al., 2019), belong to different PhaeoNet merged modules (cyan, tan, steelblue and magenta), suggesting a relatively complex regulation of this hub. The plastid-localized GS (encoded by Phatr3_J51092) belongs to the magenta merged module, which also contains the subsequent genes encoding enzymes mediating the entry of GS-produced NH_3_ into the mitochondrial ornithine-urea cycle (Phatr3_J42398 encoding malate dehydrogenase; Phatr3_J30145 encoding citrate synthase; Phatr3_J22913 encoding pyruvate kinase), suggesting this co-regulated pathway may have roles in recycling excess NH_3_ produced in the chloroplast, in accordance with previous studies (Levering et al., 2016; Broddrick et al., 2019; Smith et al., 2019).

Finally, we noted the presence of genes encoding chloroplast-targeted plastoquinol terminal oxidase (Phatr3_J4283) and mitochondria-targeted alternative oxidase (Phatr3_EG02359), which are both associated with the photoprotective removal of excess metabolic reducing potential in the skyblue merged module (Bailleul et al., 2015; Murik et al., 2019). This merged module, as discussed above, contains genes encoding three successive enzymes associated with the TCA cycle (Phatr3_J40430 encoding α-ketoglutaryl dehydrogenase; Phatr3_J42015 encoding succinyl-CoA synthetase and Phatr3_J41812 encoding succinate dehydrogenase; Kroth et al., 2008), along with methylmalonyl-CoA mutase (Phatr3_J51830), which may allow excess succinyl-CoA to be diverted into lipid synthesis via propionyl-CoA (Helliwell et al., 2011; Valenzuela et al., 2012). This co-regulation underlines the importance of the succinate hub, and presumably both the glyoxylate cycle and ornithine shunt (as sources of mitochondrial α-ketoglutarate), as routes for the mitochondrial dissipation of excess chloroplast reducing potential (Bailleul et al., 2015; Broddrick et al., 2019).

### Transcriptional Regulators of Chloroplast-Targeted Proteins Show Separate Expression Dynamics, Informed by Evolutionary History

Finally, given the complex transcriptional partitioning of genes encoding components of chloroplast and mitochondrial metabolism pathways across PhaeoNet data, we investigated what transcriptional drivers might be implicated in the co-regulation of different metabolism-enriched pathway clusters. First, we considered the repartition of a manually curated list of genes encoding proteins implied in histone and transcription-related processes (including transcription factors, TFs; Rayko et al., 2010; Banerjee et al., 2016) across all merged modules (Supplementary Figure 7 and Supplementary Table 4). These genes were most frequently observed (>5% of total merged module genes) in the darkgray, brown and steelblue merged modules (Supplementary Figure 7 and Figure 3). The brown and darkgray merged modules were additionally enriched in KEGG merged modules related to cytoskeleton proteins (Supplementary Figure 4), pointing to close links between cytoskeletal organization and transcriptional regulation in diatoms (for example, within organization of the cell cycle (Huysman et al., 2013; Tanaka et al., 2015). The single most abundant TF family, heat shock factor family (HSF) proteins (Rayko et al., 2010), were most frequently detected in the brown, brown4, cyan and skyblue merged modules (> 5 HSFs each, Supplementary Figure 7). Notably, both the brown and brown4 merged modules are also enriched in KEGG functions associated with stress responses (protein ubiquitinylation, autophagy and membrane trafficking) (Supplementary Figure 5), consistent with previously inferred functions of specific *P. tricornutum* HSFs in the maintenance of cellular fitness (Chen et al., 2014; Egue et al., 2015).

We also found specific repartitions of genes encoding proteins implicated in light- and circadian-dependent transcriptional responses in *P. tricornutum*, e.g., aureochromes and cryptochromes (Takahashi et al., 2007; Banerjee et al., 2016). These proteins typically have cytoplasmic localizations, but through the perception of light and translocation to the nucleus can regulate the expression of core chloroplast metabolic pathways (Kroth et al., 2017). The circadian-regulated Aureochrome 1c (Phatr3_J12346; Banerjee et al., 2016; Kroth et al., 2017) and a cryptochrome-like blue light receptor (Phatr3_J34592) were both found in the blue merged module, implicated in anabolic metabolism; and the light-regulated Aureochrome 1b (Phatr3_J15977) and the blue-light-dependent protochlorophyllide reductase 1 (Phatr3_J12155; Hunsperger et al., 2016; Mann et al., 2017) were both found in the lightsteelblue1 merged module, alongside the majority of genes encoding other pigment biosynthesis enzymes. In contrast, the gene encoding Aureochrome 1a (Phatr3_J49116), which is essential for high light acclimation but appears to be under exclusively circadian (light-independent) regulation, falls within the lightcyan1 merged module of core photosystem-associated genes (Supplementary Table 4 and Supplementary Figure 7; Banerjee et al., 2016; Mann et al., 2017); while RITMO1 (Phatr3_J44962), associated with the *P. tricornutum* circadian clock, falls within the skyblue merged module, which contains limited chloroplast-related functions except for alternative electron flow pathways (Supplementary Table 4 and Supplementary Figure 7; Annunziata et al., 2019). The separate distributions of light- and circadian-regulated chloroplast regulators might reflect a circadian-entrained synthesis of the core photosynthetic machinery (via Aureochrome 1a), independent of light status, with chloroplast biosynthesis pathways upregulated both by circadian signaling (via Aureochrome 1c) and as a function of light availability (via Aureochrome 1b). This is reminiscent of circadian gene expression patterns visualized in plant and other algal lineages (e.g., the green alga *Ostreococcus* and the dinoflagellate *Lingulodinium*), in which photosynthesis and plastid biogenesis proteins are either expressed at separate times of the day, or show different regulatory responses to circadian and light signals (Wang et al., 2005; Monnier et al., 2010; Noordally et al., 2013). Finally, the gene encoding the Aureochrome 2 protein (Phatr3_J8113), which lacks the conserved flavin-binding domain required for light perception (Takahashi et al., 2007; Kroth et al., 2017), falls within the greenyellow merged module of generally transcriptionally repressed proteins (Figure 3), underlining its independence of chloroplast functions.

Finally, we wished to consider within our dataset what transcriptional dynamics within the nuclear genome may underpin chloroplast gene expression in *P. tricornutum.* Chloroplast transcription in *P. tricornutum*, as in other diatoms, is performed by a plastid-encoded RNA polymerase, unlike the situation in plants in which both plastid- and nuclear-encoded and plastid-targeted polymerases participate (Oudot-Le Secq et al., 2007; Yu et al., 2018). Plastid-encoded RNA polymerases in plants typically interact with nucleus-encoded sigma factors, which may direct them to specific target genes, in response to different regulatory and physiological signals (Shimizu et al., 2010; Noordally et al., 2013). Eight genes are annotated in the *P. tricornutum* nuclear genome to encode sigma factor related proteins (Rayko et al., 2010; Supplementary Table 4), but the functions of each protein with regard to the expression of the chloroplast genome remain unclear.

**FIGURE 5:**
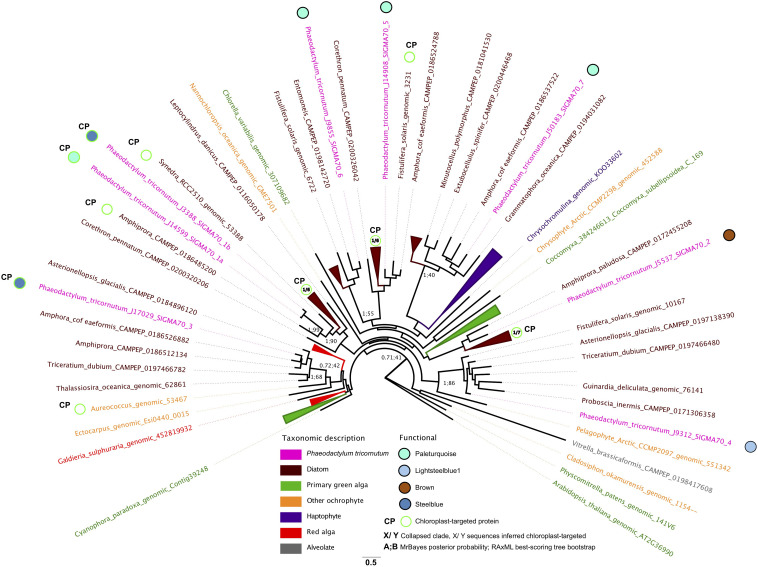
Phylogenetic and transcriptional dynamics of *P. tricornutum* sigma factors. This Figure shows an unrooted best-scoring tree topology for an 86 taxa x 453 aa alignment of subsampled diatom and non-diatom sigma factors and realized using MrBayes v 3.2.7a with the Jones substitution matrix, 600,000 generations, two start chains and 0.5 burnin thresholds (Huelsenbeck and Ronquist, 2001); and RAxML v 8.2 with the PROTGAMMAJTT substitution model with 300 bootstrap replicates (Stamatakis, 2014). Chloroplast-targeting predictions were performed using ASAFind with SignalP v 3.0 (Gruber et al., 2015); and HECTAR (Gschloessl et al., 2008) under default conditions. Branches are colored by phylogenetic affiliation and bootstrap values of nodes recovered with > 40% support are shown. Eight *P. tricornutum* sigma factors are labeled with PhaeoNet merged module repartition and chloroplast targeting sequences were predicted by HECTAR or ASAFind (Gruber et al., 2015).

We investigated the functions of *P. tricornutum* sigma factors by combining the repartition of each sigma factor in PhaeoNet with predicted *in silico* localizations of *P. tricornutum* proteins and their closest homologs from other diatom species, as resolved with a single-gene (RAxML) tree (Figure 5). Three of the sigma factor genes in *P. tricornutum* possess chloroplast-targeting sequences, as inferred by *in silico* prediction with HECTAR and ASAFind (Gschloessl et al., 2008; Gruber et al., 2015). One of these proteins (Phatr3_J14599, SIGMA1a) falls within the paleturquoise merged module, which is otherwise enriched in chloroplast-related functions pertaining to carbon concentration and the glycine shunt (Figures 3–5); while the two remaining chloroplast-targeted proteins (Phatr3_J3388, SIGMA1b; Phatr3_J17029, SIGMA3) fall within the steelblue module, which otherwise lacks obvious enrichments in chloroplast-targeted functions and instead seems to be most closely connected to nucleotide metabolism (Figures 3, 5). Phylogenetic analysis of these three sigma factors indicate that many of their closest relatives are sequences with chloroplast-targeting signals from other diatoms, and indeed SIGMA1a and SIGMA1b appear to be recently derived paralogs of one another (Figure 5), indicating that they are likely to be conserved parts of the diatom chloroplast transcriptional machinery. The repartition of SIGMA1b and SIGMA3 within a transcriptional module that is largely related to non-chloroplast processes may allow hierarchical control of chloroplast transcription in response to non-chloroplast signals in *P. tricornutum* (e.g., coordination with circadian or cell cycles, Noordally et al., 2013; Tanaka et al., 2015).

The remaining five *P. tricornutum* sigma factors were not predicted to be targeted to the chloroplast and phylogenetic analysis indicated that their closest diatom relatives primarily also lacked chloroplast-targeting signals (Figure 5). One of these non-chloroplast-associated sigma factors (Phatr3_J5537, SIGMA2) fell within the largely chloroplast-independent brown module, suggesting that it has non-chloroplastic functions. In contrast, the remaining non-chloroplast targeted sigma factors fell within modules otherwise enriched in chloroplast-associated functions; either lightsteelblue (Phatr3_J9312, SIGMA4), or paleturquoise (Phatr3_J14908, Phatr3_J9855, Phatr3_J50183; SIGMA 5-7; Figures 3, 5). It remains to be determined whether these sigma factors are targeted to the *P. tricornutum* chloroplast, but using alternative methods to those recognized by HECTAR or ASAFind, as per certain other diatom proteins (Kazamia et al., 2018; Schober et al., 2019); function in compartments other than the chloroplast, but participate indirectly in the regulation, e.g., of nucleus-encoded proteins implicated in chloroplast metabolism; or have functions independent of the chloroplast, as has been documented for some other eukaryotic sigma factors (Shadel and Clayton, 1995; Beardslee et al., 2002). These different possibilities may be best discriminated by the experimental characterization, e.g., through mutagenesis and functional phenotyping, of individual *P. tricornutum* sigma factor genes.

## Concluding Remarks

In this project, we have used WGCNA to build an integrated network of *P. tricornutum* gene co-regulation, which we name “PhaeoNet.” Our model is able to retrieve well established biological pathways (e.g., chloroplast photosynthetic, anabolic metabolism; and mitochondrial respiration, Figure 4) and compares favorably to existing (e.g., microarray-based; Ashworth et al., 2016) studies of gene co-regulation for this species (Ashworth et al., 2016; Figures 1, 2 and Supplementary Figures 1–4). Moreover, our dataset carries the advantage of decomposing the *P. tricornutum* genome into a smaller number (28) of functionally distinct modules than produced by DiatomPortal. We have integrated these data into previously generated functional, targeting and evolutionary analyses of the *P. tricornutum* genome, allowing us to gain holistic insights into the processes underpinning the gene co-regulation of specific biological processes and organelle metabolic pathways pertinent to diatom biology (Figure 3 and Supplementary Figure 5).

Through a deeper inspection of genes encoding chloroplast and mitochondria-targeted proteins within these data, we identify PhaeoNet merged modules underpinning anabolic (blue), photosynthetic (lightsteelblue 1, lightcyan1) and respiratory (orangered4, cyan) metabolism, and identify multiple metabolic connections between the chloroplast and mitochondria. These include the glycine shunt within the paleturquoise merged module; the ornithine-urea cycle within the magenta merged module; and coordinated chloroplast and mitochondrial alternative oxidase activities in the skyblue merged module; Figure 4 and Supplementary Figure 6. Finally, considering the repartition of transcription-related proteins within our data, we identify probable cognate regulators for different co-ordinated metabolic pathways (Figure 5 and Supplementary Figure 7), demonstrating different associations of aureochrome transcription factors with different chloroplast metabolic pathways. We notably identify hidden diversity in the range of sigma factor genes in the *P. tricornutum* genome, some of which are likely to be involved in the transcriptional regulation of different chloroplast-encoded genes in response to different physiological signals, while others are likely to have different functions to chloroplast gene expression.

The PhaeoNet dataset may be usable as a predictive tool for the characterization of poorly understood proteins, either directly in *P. tricornutum*, as a well-studied model diatom species, or in other diatom or microalgal species for which homologs of *P. tricornutum* proteins are known either from genome or transcriptome datasets (e.g., Keeling et al., 2014; Carradec et al., 2018; Sato et al., 2020). We stress that biological processes elucidated in this species may not necessarily be directly extrapolatable to other algal species; and examples are already known of proteins (e.g., proteins involved in iron-stress tolerance and C4 photosynthesis) that may have different physiological functions even between different diatoms (Kustka et al., 2014; Lampe et al., 2018). Cross-comparisons between PhaeoNet and other data, e.g., gene coregulation datasets erected in other, less well-studied species (Ashworth et al., 2016; Ashworth and Ralph, 2018); environmental expression trends (Carradec et al., 2018); and the phenotypes of a wider range of mutant lines generated in *P. tricornutum* will be essential to understanding the diversity of functions performed by understudied proteins in diatoms and other algae. Nonetheless, insights from our data, delivering actors and signatures of metabolic co-regulation in diatoms, will provide a useful community resource for subsequent directed experimental investigation.

## Data Availability Statement

Publicly available datasets were analyzed in this study. This data can be found here: https://osf.io/42xmp/.

## Author Contributions

OA-M was responsible for the design and construction of PhaeoNet. AMGNV and NJ performed the functional analysis of the PhaeoNet modules. YL and XZ participated in the construction of the data used for functional analysis. AG, LT, and CB were responsible for the supervision of the construction of PhaeoNet. RGD was responsible for the supervision of functional analysis. OA-M and RGD wrote the manuscript, with input from all other co-authors. All authors contributed to the article and approved the submitted version.

## Conflict of Interest

The authors declare that the research was conducted in the absence of any commercial or financial relationships that could be construed as a potential conflict of interest.

## Footnotes

1 http://www.ncbi.nlm.nih.gov/Traces/sra/

2 https://www.bioinformatics.babraham.ac.uk/projects/fastqc/

3 http://www.bioinformatics.babraham.ac.uk/projects/trim_galore/
