# Supplementary figures and images for: PhaeoNet: A Holistic RNAseq-Based Portrait of Transcriptional Coordination in the Model Diatom Phaeodactylum tricornutum
Source: Front Plant Sci. 2020 Oct 16;11:590949. doi: 10.3389/fpls.2020.590949 (PMC7596299; doi:10.3389/fpls.2020.590949)

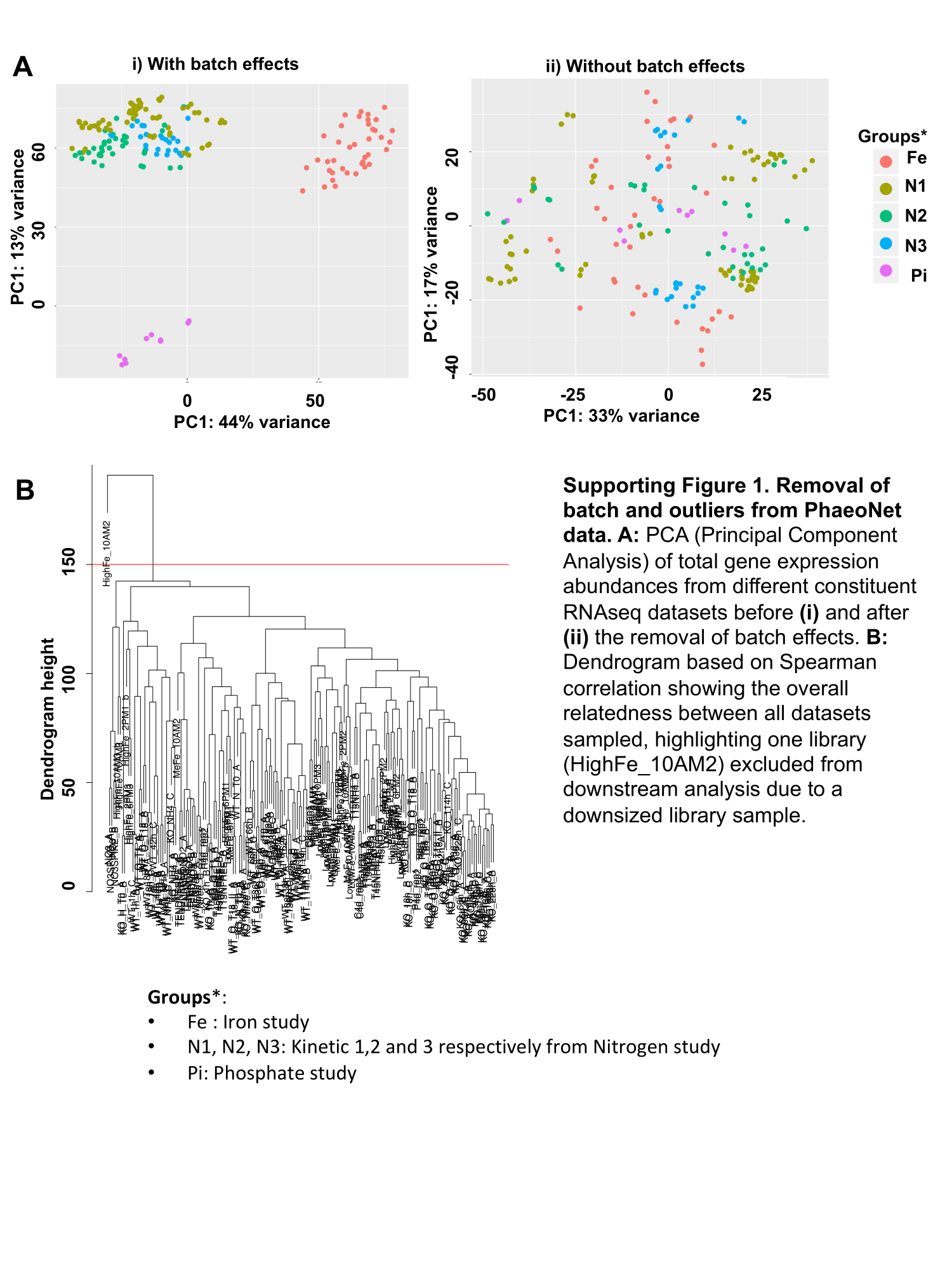

Supplement: Supplementary file 6 [file Image_1.TIFF]

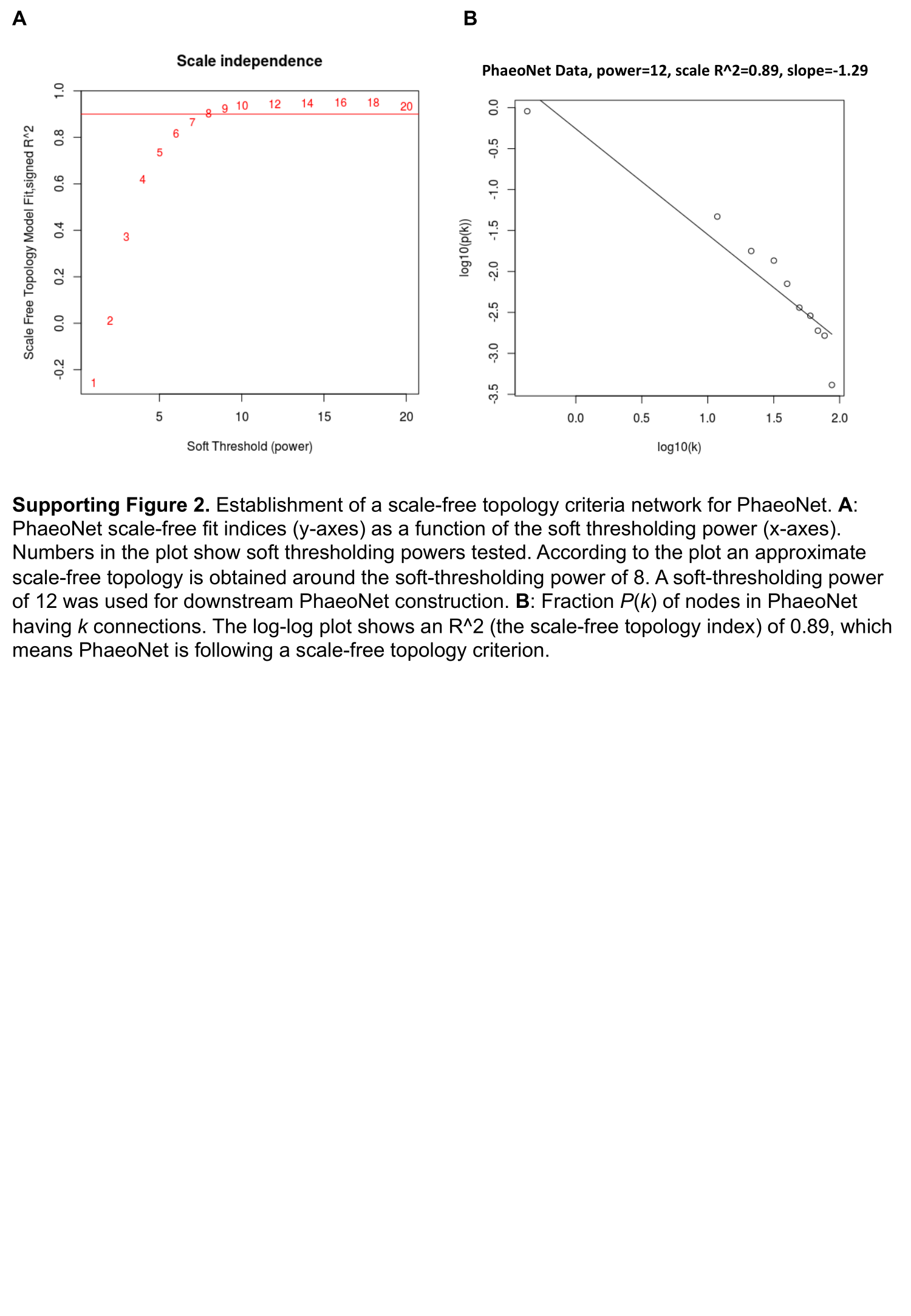

Supplement: Supplementary file 7 [file Image_2.TIFF]

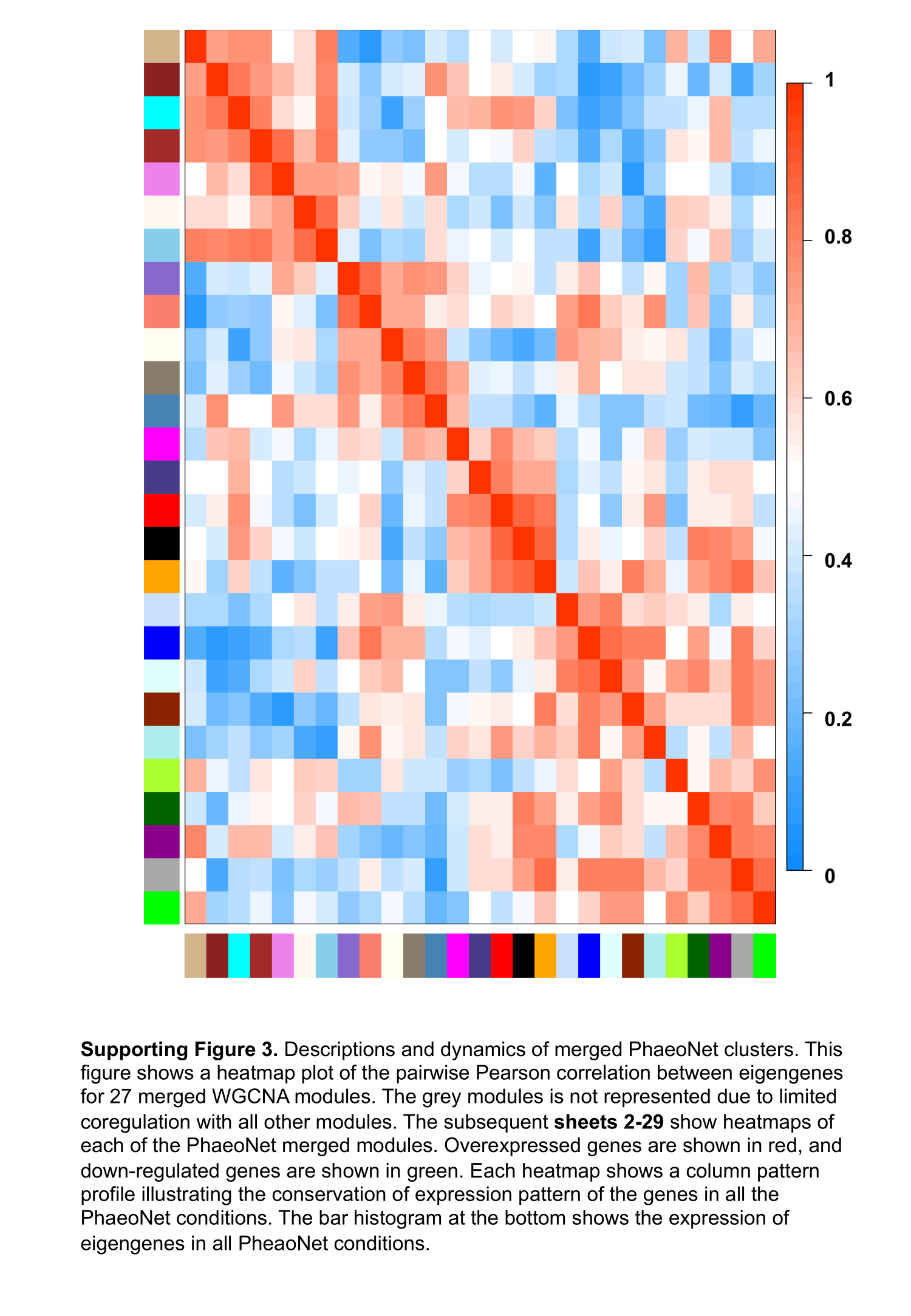

Supplement: Supplementary file 8 [file Image_3.TIFF]

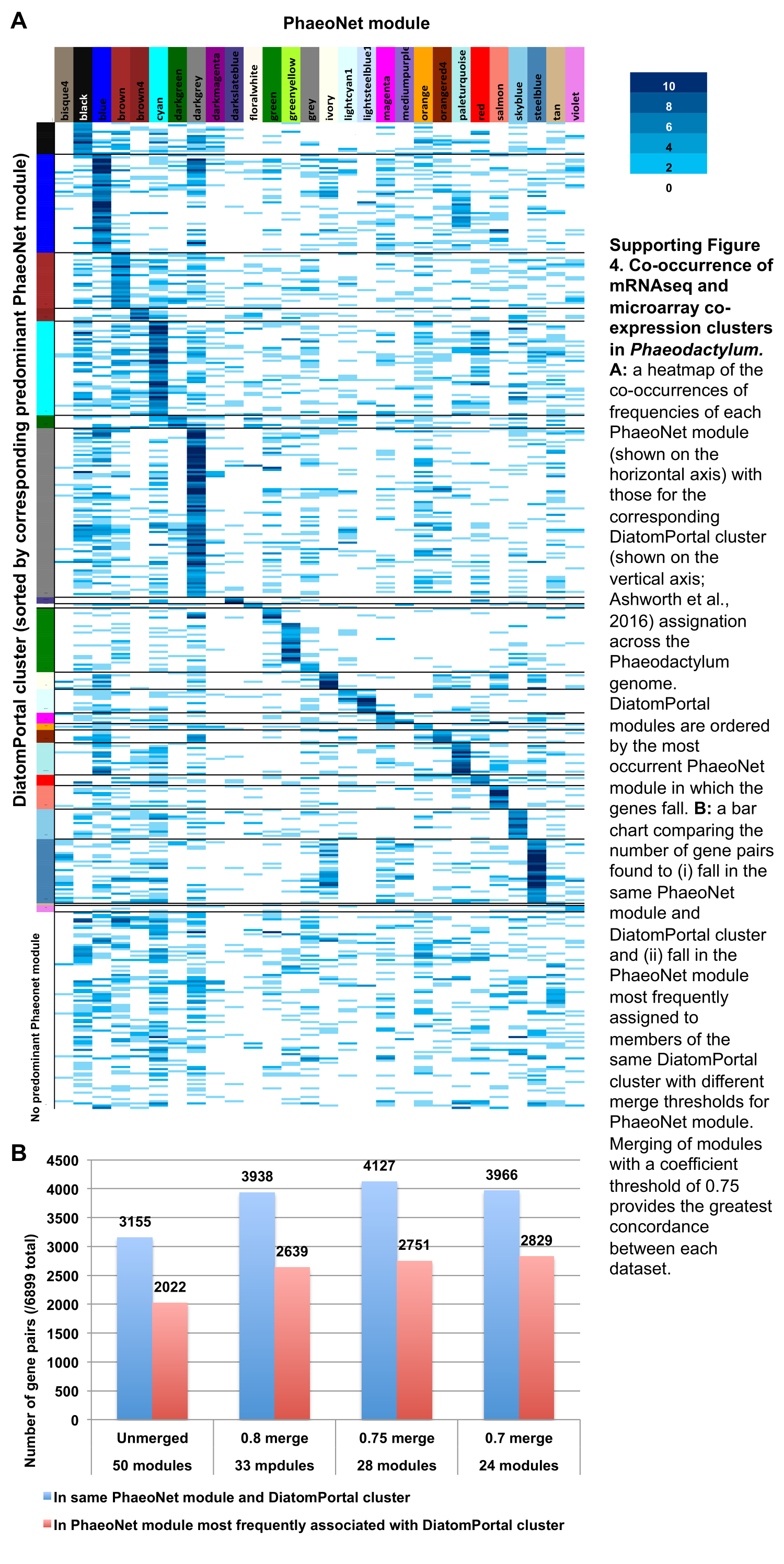

Supplement: Supplementary file 9 [file Image_4.TIFF]

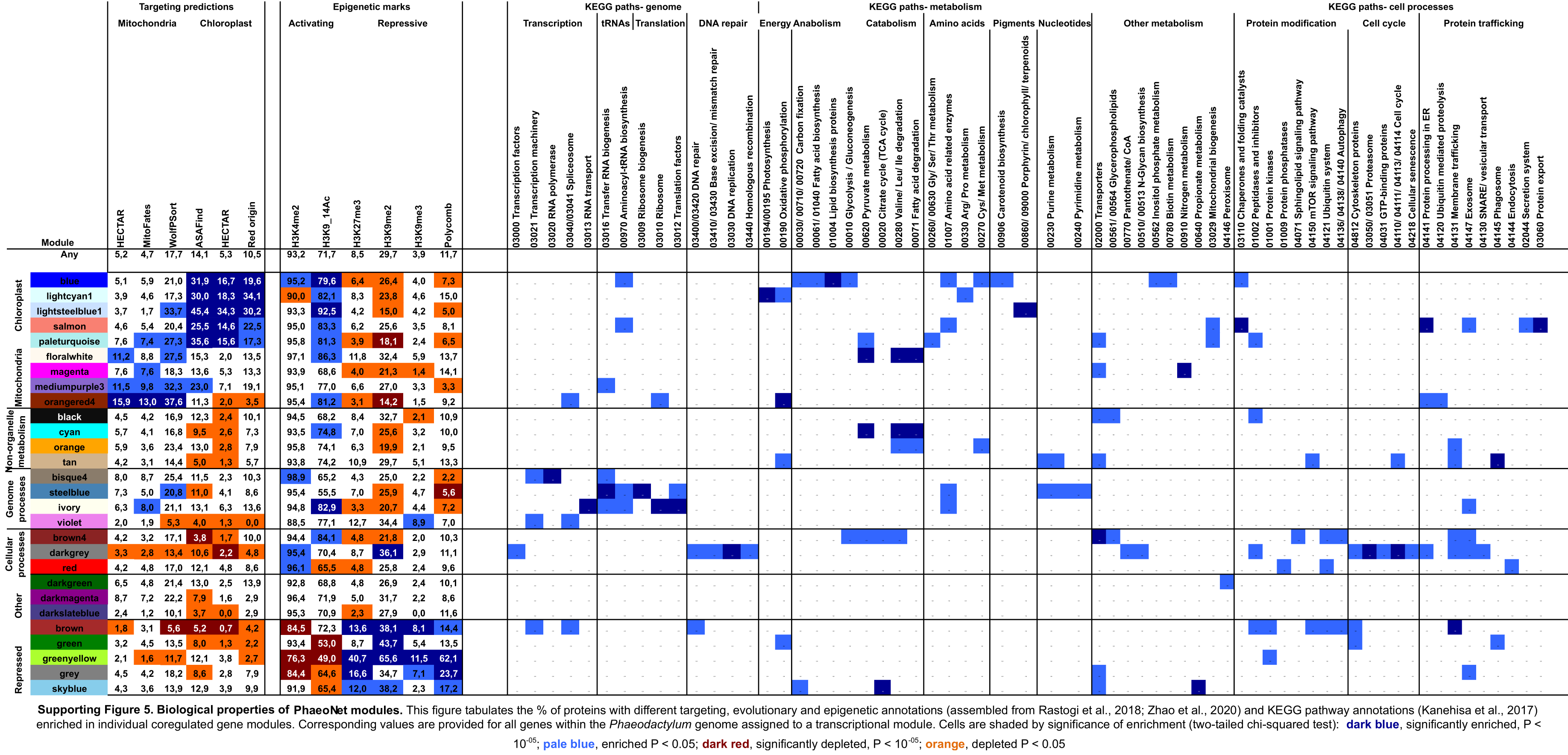

Supplement: Supplementary file 10 [file Image_5.TIFF]

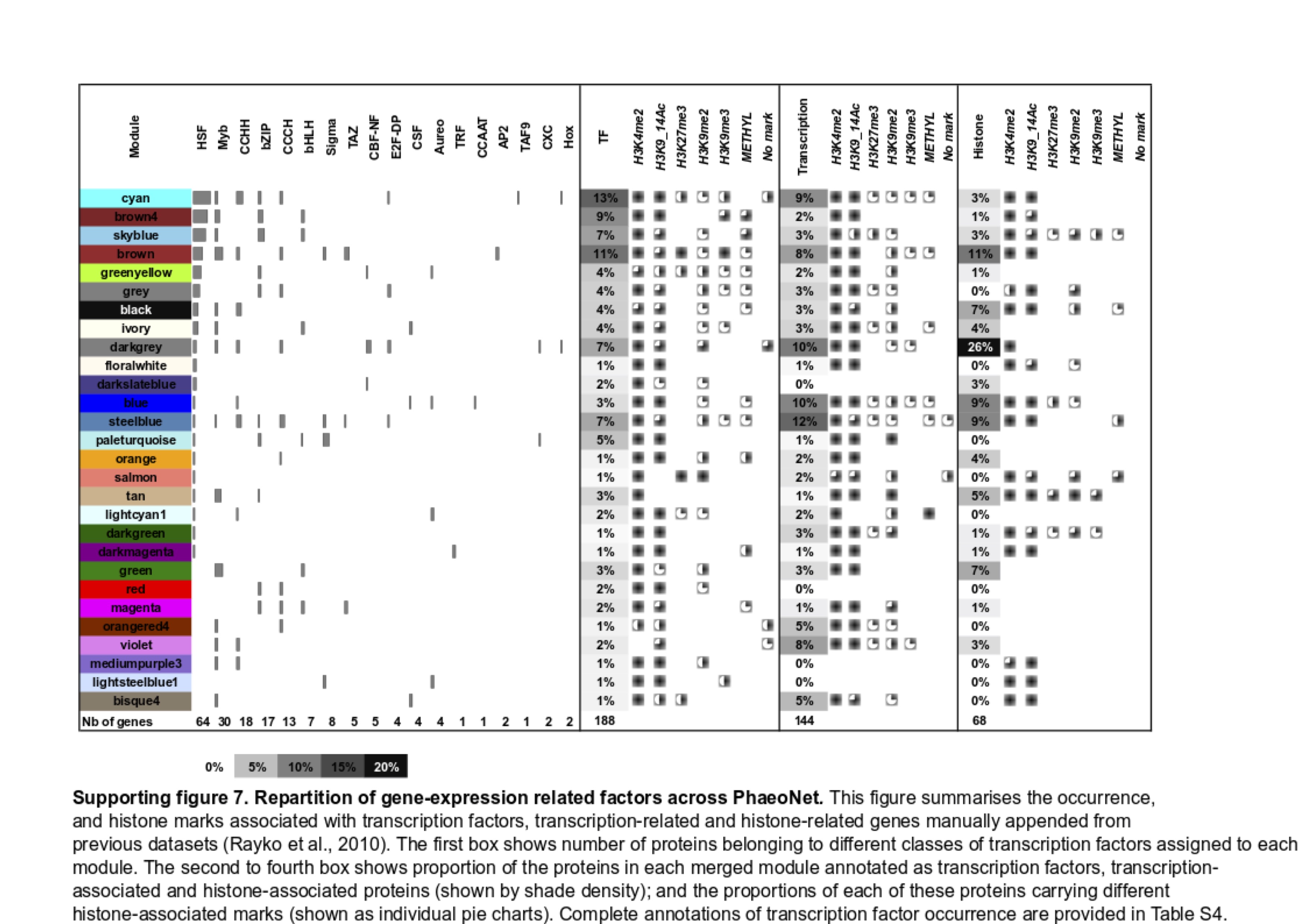

Supplement: Supplementary file 12 [file Image_7.JPEG]
